# P-1365. Cefazolin Inoculum Effect in Methicillin Susceptible *Staphylococcus aureus* Recovered From the Nasal Cavity of Patients in Intensive Care Units in Colombia

**DOI:** 10.1093/ofid/ofae631.1542

**Published:** 2025-01-29

**Authors:** Sandra Rincon, Lina Paola Carvajal, Laura Ibarra, Maria José Cadavid, Mauricio Uribe, Juan Matiz, Karen M Ordonez Diaz, Fredy Guevara-Pulido, Alexandra Porras, Leonardo Ospina, Yardany Mendez, Haley Greenia, Rodrigo de Paula Baptista, Cesar A Arias, Jinnethe Reyes

**Affiliations:** Molecular Genetics and Antimicrobial Resistance Unit and International Center of Microbial Genomics, Universidad El Bosque, Bogota, Colombia, Bogota, Distrito Capital de Bogota, Colombia; Universidad El Bosque, Bogotá D.C., Distrito Capital de Bogota, Colombia; Universidad El Bosque, Bogotá D.C., Distrito Capital de Bogota, Colombia; Universidad El Bosque, Bogotá D.C., Distrito Capital de Bogota, Colombia; Universidad El Bosque, Bogotá D.C., Distrito Capital de Bogota, Colombia; Universidad el Bosque, Bogotá, Distrito Capital de Bogota, Colombia; Grupo de Investigación Hospital Universitario San Jorge, Pereira, Risaralda, Colombia; Clínica Reina Sofía, Bogota, Distrito Capital de Bogota, Colombia; Los Cobos Medical Center, Bogotá D.C., Distrito Capital de Bogota, Colombia; Hospital de Mederi, Bogotá, Cundinamarca, Colombia; Hospital Regional de Duitama, Duitama, Boyaca, Colombia; Houston Methodist Research Institute, Houston, Texas; Houston Methodist Hospital, Houston, Texas; Houston Methodist and Weill Cornell Medical College, Houston, TX; Molecular Genetics and Antimicrobial Resistance Unit, Universidad El Bosque, Bogota, Distrito Capital de Bogota, Colombia

## Abstract

**Background:**

Cefazolin is an alternative for the treatment of MSSA infections and first choice option for surgical prophylaxis. The cefazolin inoculum effect (CzIE), the increase in the MIC at high bacterial inocula is associated to therapeutic failures and increased mortality in deep-seated MSSA infections. The prevalence of the CzIE among nasal colonizing MSSA from ICU patients is currently unknown. The aim of this study was to investigate the CzIE in nasal MSSA recovered from patients admitted to ICUs in Colombia.

**Methods:**

We evaluated 33 MSSA isolates recovered from nasal swabs of 29 patients admitted to ICU at 6 high-complexity Colombian hospitals (2019-2023). CFZ MIC using gold standard broth microdilution at high inoculum was done. The modified nitrocefin-based rapid test to identify the CzIE was performed, and diagnostic performance metrics were calculated. Whole genome sequencing was used to characterize BlaZ types and allotypes, Clonal Complexes CC and Accessory Gene Regulator (Agr) types.

**Results:**

The CzIE was identified in 54.5% of nasal MSSA isolates. A high prevalence of nasal colonization with MSSA exhibiting the CzIE was detected (59%, 17 patients). Among 18 MSSA with the CzIE, type A BlaZ was the predominant β-lactamase (61%) and BlaZ-2 was the most common allotype (50%). Whereas among 15 MSSA lacking the CzIE, BlaZ type C was the most frequent (47%) and BlaZ-1 was the allotype predominant (40%). A high diversity of genetic lineages with eight CC was detected among the 33 MSSA. CC30 was the predominant lineage in MSSA displaying the CzIE (44%), while CC5 and CC30 (27% each one) were the most common in MSSA without the CzIE. Agr-III and Agr-I were predominant Agr types in 56% and 47% of MSSA with the CzIE and lacking the CzIE, respectively. Compared to the gold standard the modified rapid test identified nasal MSSA isolates showing the CzIE with a sensitivity of 100%, specificity of 93% and an overall accuracy of 97%.

**Conclusion:**

We found an unexpected high prevalence (59%) of ICU patients colonized by MSSA exhibiting the CzIE, showing similar genetic features to MSSA with CzIE from invasive infections. Furthermore, our findings suggest that the nasal cavity of ICU patients may represent a reservoir for the dissemination of MSSA showing the CzIE, highlighting the importance of screening and surveillance.

**Disclosures:**

**All Authors**: No reported disclosures

